# *Thirsty? Choose Water!* A regional perspective to promoting water consumption in secondary school students

**DOI:** 10.1017/S1368980023001313

**Published:** 2023-11

**Authors:** Nicole Kajons, Justine Gowland-Ella, Samantha Batchelor, Nina Kingon, Michael David

**Affiliations:** 1 Health Promotion Service, Central Coast Local Health District, Gosford, NSW 2250, Australia; 2 School of Medicine and Dentistry, Griffith University, Gold Coast, QLD 4222, Australia; 3 The Daffodil Centre, The University of Sydney, a joint venture with Cancer Council, NSW 153, Dowling St, Sydney, NSW 2011, Australia

**Keywords:** Randomised controlled trial, Health-promoting schools, School intervention, Obesity, Water consumption, Sugar-sweetened beverages, Chilled water stations

## Abstract

**Objective:**

Adolescents are high consumers of sugar-sweetened beverages (SSB), which contribute to overweight and obesity – a significant public health issue. Evidence suggests that replacing SSB with water and school-based interventions can reduce consumption. This study examines the acceptability of a previously trialled intervention (Thirsty? Choose Water!) in regional and remote secondary schools.

**Design:**

An open-label randomised controlled trial using a two-by-two factorial design tested the outcomes of a behavioural and/or environmental intervention on SSB and water consumption.

**Setting:**

Regional and remote secondary schools (public, catholic and independent) within the boundaries of two regional Local Health Districts within New South Wales.

**Participants:**

Twenty-four schools participated in the study. The target group was year 7 students (*n* 1640) – 72 % of eligible students completed baseline data. The study followed students into year 8 (*n* 1188) – 52 % of eligible students completed post-intervention data. Forty teachers undertook training to deliver the intervention.

**Results:**

Interventions showed high levels of acceptability. Students demonstrated changes in knowledge, attitudes and consumption behaviours. Multivariable ordinal logression analysis demonstrated that all interventions increased the odds of students increasing their water consumption (though not statistically significant). Conversely, the combined (OR: 0·75; 95 % CI: 0·59, 0·97) or environmental intervention (OR: 0·68; 95 % CI: 0·51, 0·90) had greater odds of reducing SSB consumption and was statistically significant.

**Conclusions:**

This study builds on recent Australian evidence regarding the impact of school-based interventions on water and SSB consumption. In this study, despite a minor intervention change, and the impacts of fires, floods and COVID-19 on study implementation, the interventions were highly regarded by the school communities with positive outcomes.

Children and adolescents are high consumers of sugar-sweetened beverages (SSB) (soft drinks, energy drinks, fruit drinks, sports drinks and cordial) which are energy-rich, providing almost no nutritional value^(1)^. In Australia, consumption increases with age: 60 % of 12–15-year-olds regularly consume SSB compared with 36·4 % of 5–11-year-olds^(2)^. Among 13–14-year-olds, 10·4 % report at least daily consumption, with 30 % reporting consuming 2–6 cups per week^(3)^. This is concerning as firm evidence links SSB consumption to various health issues in children and adolescents. Most prominent is their contribution to overweight and obesity^(4)^, but they are also contribute to dental caries^(5)^, insulin resistance^(4)^, metabolic health issues^(6)^, aggressive behaviours^(7)^, and poor sleep, risk-seeking behaviour and depressive symptoms (linked to caffeinated energy drinks)^(4)^.

Whilst a range of higher-level policy options to address SSB consumption are emerging, including sugar taxes^(8)^ and restrictions on SSB marketing^(9)^, evidence also suggests that substituting SSB with water can have a positive effect^(10,11)^. Choosing water as a drink may reduce daily energy consumption and positively impact obesity, BMI^(10)^ and dental caries. Children who are well hydrated at school also have better attention, memory, cognition and learning^(12–14)^. However, children’s water consumption is often below-recommended levels^(15)^. Many children arrive at school dehydrated, do not drink adequate amounts throughout the school day and consume only 14 % of their total fluid intake at school^(16)^. Additionally, research suggests that while water quality in regional New South Wales (NSW) is generally acceptable, it is the temperature of water that is most problematic where water bubblers are unpopular, especially for children in summer, when the surface temperature of steel bubblers can reach above 50°C^(17)^.

Growing research has explored how to decrease SSB consumption and promote water consumption in school settings. Recent reviews of school-based interventions addressing SSB consumption show a positive impact with a trend towards reducing SSB consumption^(18,19)^, whether they target the individual, the school environment or both^(20)^. Similarly, positive effects were also shown in interventions promoting water with a combined approach using educational/behavioural and legislative/environmental components holding the most promise^(21)^, while water promotion interventions that alone were shown not decrease SSB consumption^(22)^. Citizen science utilising the school student body^(23)^, social networking interventions utilising peer approaches^(24)^, and teachers as role models have all been trialled interventions to increase raise awareness of the need for appropriate water sources and promote healthy drink choices.

In Australia, school-based health promotion activities have focused on nutrition, physical activity, canteens, changes in the school environment or a combination of these^(25–27)^. However, *‘*Thirsty? Choose Water! is the first Australian research to solely address SSB and the promotion of water, by testing a behavioural and environmental intervention in secondary schools. The research followed a translational research approach^(28)^. Grant funding adapted and expanded the pilot study for implementation in sixty-one secondary schools in the Greater Sydney region^(29)^. Positive outcomes in reducing SSB consumption and increasing water consumption were demonstrated^(30,31)^. Subsequently, further funding was acquired to translate the interventions to regional and remote school settings. This paper reports on this second study, examining the viability and acceptability of the two interventions (alone or combined) in regional/remote schools and the impacts on students’ knowledge and consumption behaviours (including the primary outcome of increasing water consumption and the secondary outcome of decreasing SSB consumption).

It was hypothesised that consumption of water and SSB among students with access to one or both interventions in a school setting would significantly increase and decrease respectively, when compared with students without access to either intervention.

## Methods

### Study design and participants

The study examined the transferability and acceptability of the intervention(s) in regional/remote secondary schools within two NSW Local Health Districts (LHD) and the outcomes of the intervention(s) on year 7/8 (aged 12–14 years) students’ water and SSB consumption. NSW, located on the east coast of Australia, is the country’s most populous state, with Sydney being the state capital.

Reflecting the initial study^(29)^, the regional study also used a two-by-two factorial randomised control trial design. The study adhered to the CONSORT reporting guidelines, and a checklist is included as a supplementary file. The study interventions (one behavioural and one environmental) were delivered either alone or combined. Where possible the regional study adhered to the original study protocol^(29)^, with relevant intervention adaptations made to accommodate regionality and the impacts of the evolving COVID-19 pandemic.

Eligible schools included ‘regional’ or ‘remote’ secondary schools within the boundaries of the study LHD^(32,33)^ and catholic schools within the diocese that provided ethical clearance. Schools already involved in health promotion research; schools for special purposes (e.g. for students with behavioural difficulties) or schools with adequate chilled water stations (CWS) *in situ* (deemed as one per 300 students) were excluded. Subsequently, sixty-nine schools were invited to participate – twenty-four school principals consented to their schools’ involvement. Consent was collected by the project officers. Participating schools were randomly allocated to one of four study arms by the study statistician using a computerised random number generator. Following allocation, schools completed baseline data collection (September 2019, term 3). The power and sample size calculation indicated that 2176 year 7 students were required for recruitment to the study to detect a change of 15 % or more in the primary outcome measure increased water consumption. Due to funding time frames, the study ran across school years (NB NSW school years generally run from late January to mid-December, over four school terms) – baseline data were collected from students in the latter half of year 7 (2019), whilst the behavioural intervention (BI) was conducted as the students moved into year 8 (2020) with follow-up data subsequently collected.

Ethics approval was obtained through the Hunter New England Human Research Ethics Committee (17/08/16/4.07), the NSW Department of Education (State Education Research Applications Process – SERAP2017457) and one of three Catholic Diocese within these regions.

### Intervention components

Where possible, the regional study interventions emulated the initial study (described elsewhere)^(30)^ However, funding time frames, vast distances between schools and the impacts of COVID-19 necessitated adaptation of BI components. This intervention initially consisted of three components aligning with the Health Promoting Schools framework^(34)^ – the delivery of the Thirsty? Choose Water! messages through a teaching intervention, promotional materials across the school and via the year 7 school-based vaccination programme (delivering messages during the second vaccination clinic). Given the logistics of funding timelines not aligning with vaccination programme schedule, a pragmatic decision was made to incorporate the resources used within this component into the teaching component.

The teaching intervention aligns with the NSW Stage 4 Personal Development, Health and Physical Education (PDHPE) curriculum (described elsewhere^(30)^). Two lessons delivered in PDHPE use learning activities, including videos, PowerPoint presentations, a water challenge and the ‘spouts and straws’ game (initially used in the vaccination intervention) to educate students on the benefit of water and the detrimental effects of SSB. An online self-paced learning module was developed to up-skill teachers for intervention delivery in the regional study. This training included three modules covering adolescent obesity statistics, information about SSB, the importance of drinking water and oral health benefits, lesson ideas and study implementation requirements. Within one LHD, some schools were more closely located to one another and a hybrid model including face-to-face and/or online training was used.

COVID-19 also impacted intervention delivery. Whilst many schools implemented the intervention early in 2020, some schools were either mid-way through delivery or had not commenced when NSW experienced its first COVID-19 lockdown. By 30 March 2020, homeschooling commenced for all students, unless their parents were front-line workers. This continued for approximately 8 weeks^(35)^ and demanded a rapid response from the study team to translate lessons to an online format to ensure continued intervention implementation.

The promotional intervention targeted the whole school community and reinforced messages that students received in PDHPE. This component utilised school newsletters, social media, posters and promotional materials including hi-vis vests worn by teachers and posters/flip charts at the canteen to promote the ‘choose water’ message. Schools received a content package to disseminate messages through their school community.

The environmental intervention included the installation of one CWS in each school allocated to this intervention. The same product was used as in the initial study. These stations had a high level of acceptability to schools and were sturdy and vandal-resistant. The CWS was a stainless-steel wall-mounted station with a chilled drinking fountain, bottle refill capacity and bottle refill counter. Health Promotion Officers (HPOs) in consultation with schools identified the most suitable location for the CWS. This was based on issues such as power and water supply, adequate drainage and with good access for year 7/8 students (i.e. not in a senior only area). Based on these factors, most schools located their CWS either near the canteen, near existing bubblers, in main quadrangle or thoroughfares. Following baseline data collection, CWS installation was completed, and a large promotional sticker was affixed to promote the use of the CWS.

### Data collection

To ensure uniformity between the initial and regional study, data collection tools and methods remained consistent wherever possible. Data were collected at the student and school levels and via logging of water flow through the CWS.

#### School-level data

PDHPE teachers provided feedback on the acceptability and usefulness of the online teacher training module and the lesson intervention. A key school contact completed a school information summary at baseline and follow-up, which included questions about how water consumption was promoted through the school. Like the student survey, it was amended for simplicity based on lessons learnt from the first study.

#### Student-level data

While the initial study collected student data at three time points, study timelines permitted only baseline and post-intervention data collection within the regional study. The survey ascertained changes in knowledge from the BI, including the effects of SSB and dehydration. Students were also asked about their water consumption, whether they took a drink bottle to school, where they filled it and bubbler usage. Validated questions from the NSW School Physical Activity and Nutrition Survey (SPANS)^(3)^ regarding student’s beverage consumption, where they purchased beverages and how often SSB were available in their home were also included. The initial study analysis demonstrated that the SPANS question of beverage consumption was not sensitive to detecting changes in water consumption. It used a Likert scale with the highest value that students could report drinking being two or more cups per day. To address this, an additional question was added ‘how many cups of water do you drink a day?’ with response options of the numbers 0 through to 10 or more. To add clarity and minimise misclassification to questions regarding bubblers and water stations, photos were added. Based on the analysis of open-ended questions in the initial study about reasons for not using the school bubblers, forced-choice options were included. Surveys were completed via an online survey platform, either in the classroom or at home as dictated by the impacts of COVID-19. The intention was for the post-survey to be completed approximately 2 weeks after lesson component delivery.

#### Water flow measurement

All CWS had data loggers fitted to remotely capture water flow in real time. Data were downloaded to an online portal that researchers and schools could access. Some issues were experienced with the functionality of the data loggers, and addressing these issues swiftly was difficult due to the distances between schools.

### Procedures

A Human Research Ethics Committee approved all study procedures, and relevant educational ethical clearances were attained. School principals provided consent at the school level for their schools’ participation. Parents could provide written documentation to the school if they did not wish their child to participate in the student surveys. Schools within each LHD had access to a designated project officer who guided them through the study, provided information on study processes and time frames, encouraged teachers to complete the online training, and facilitated the installation of CWS.

### Statistical analyses

All statistical analyses were conducted with Stata V16.0 (StataCorp). Descriptive statistics at the school and student level were calculated for relevant variables by frequency counts and percentages. Bar charts were used to visually compare teacher feedback on the various teaching components and promotional materials, whilst a time-frequency graph was used to show actual water consumption as measured by the data loggers.

Ordinal logistic regression modelling was used to estimate the marginal and joint effects of BI and CWS on the outcomes of weekly consumption of plain water and SSB. We controlled for correlation between baseline and follow-up using clustered standard errors at the student level. In doing so, our analysis avoided using a complete-case analysis approach, as students who completed only one of the two surveys were not excluded from the analysis. To control for confounding and ensure all models were parsimonious, a two-stage screening process was undertaken. Variables with a univariable *P*-value < 0·05 were further assessed for model inclusion using a manual stepwise backward elimination approach (*P* ≥ 0·05 for removal).

For each analysis, unadjusted and adjusted OR with 95 % CI were calculated for all retained variables. The assumption of proportional hazards and model specification were assessed by the Brant test^(36)^(Brant, 1990) and the Pregibon link test^(37)^, respectively.

## Results

**Fig. 1:**
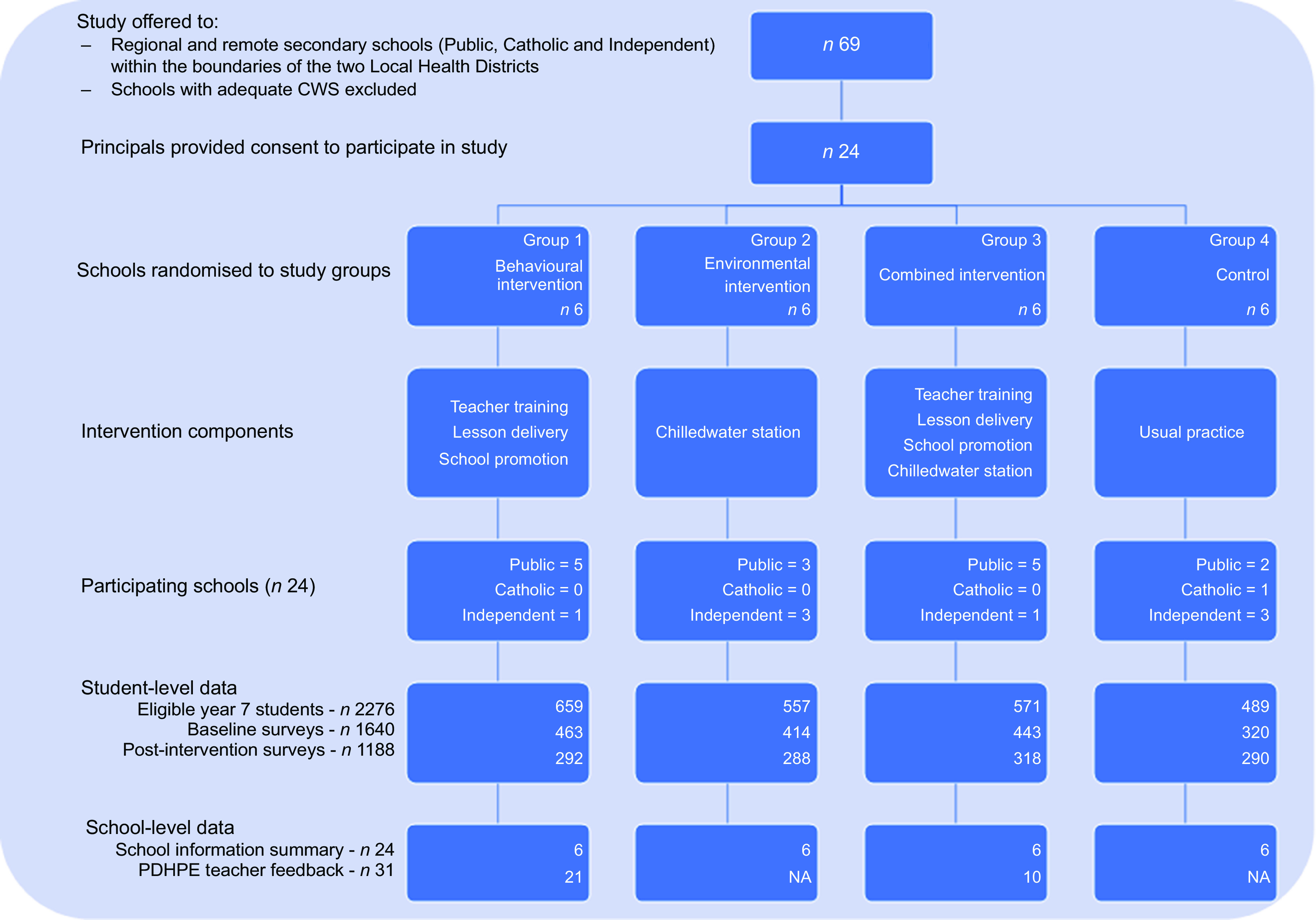
Flowchart of Thirsty? Choose Water! – schools and students. CWS, chilled water stations

Figure 1 shows school recruitment and counts of students participating in data collection. Sixty-two per cent were government, and 33 % were independent schools. Only one catholic school participated, given only one of three dioceses within the study boundaries provided ethical clearance for participation. At baseline, 72 % (*n* 1640) of eligible students (*n* 2276) completed data collection, reducing to 52 % post-intervention. Table 1 provides school-level and student-level characteristics. There were no statistically significant differences between study groups at the school level; however, at the student level, statistically significant differences were noted with a high proportion of Aboriginal and Torres Strait Islander students in groups 1 and 3 (18·66 % and 19·09 %, respectively) compared with groups 2 and 4 (11·44 % and 14·11 %, respectively) and a much lower proportion of students from a non-English-speaking background in group 4 (1·58 %) compared with the highest number in group 2 (8·62 %). Similarly, group 4 also had the lowest proportion of female students (41·56 %) compared with the highest proportions in groups 1 and 2 (51·62 and 51·69 %, respectively).

**Table 1:** School-level (*n* 24) and student-level (*n* 1640) characteristics across groups at baseline

School-level characteristics	Behavioural intervention (*n* 6)		Environmental intervention (*n* 6)		Combined intervention (*n* 6)		Control (*n* 6)		*P*-value†
	*n*	%	*n*	%	*n*	%	*n*	%	
Designated year 7 playground									
No	3	50	2	33	4	67	2	33	0·81
Yes	3	50	4	67	2	33	4	67	
Distance to nearest shop									
< 500 m	5	83	3	50	1	17	1	17	0·24
500 m–999 m	1	17	2	33	2	33	3	50	
1000 m+	0	0	1	17	3	50	2	33	
LHD									
LHD-1	2	33	2	33	3	50	4	67	0·81
LHD-2	4	66	4	67	3	50	2	33	
School canteen									
Parent- or school-operated	6	100	4	67	6	100	3	50	0·11
Neither	(0)		2	33	0	0	3	50	
School type									
Public	5	83	3	50	5	83	2	33	0·28
Private	1	17	3	50	1	17	4	67	
ICSEA*									
< 965	4	67	3	50	5	83	1	17	0·43
965–1014	1	17	2	33	1	17	3	50	
1015+	1	17	1	17	0	0	2	33	
Vending machines									
No	6	100	6	100	6	100	5	83	1·00
Yes	0	0	0	0	0	0	1	17	
Chilled water stations									
No	3	50	4	67	6	100	4	67	0·39
Yes	3	50	2	33	0	0	2	33	
Water consumption promoted									
No	0	0	3	50	4	67	3	50	0·12
Yes	6	100	3	50	2	33	3	50	
Water bubblers									
< 10	2	33	3	50	4	67	4	67	0·80
10–19	2	33	1	17	1	17	2	33	
20+	2	33	2	33	1	17	0	0·00	

	Behavioural intervention (*n* 463)		Environmental intervention (*n* 414)		Combined intervention (*n* 443)		Control (*n* 320)		
Student-level characteristics	*n*	%	*n*	%	*n*	%	*n*	%	*P*-value†
**Aboriginal and/or Torres Strait Islander									
No	376	81	365	89	356	81	274	86	0·00
Yes	86	19	47	11	84	19	45	14	
**English is the primary language spoken at home									
Yes	431	95	371	91	412	95	312	98	0·00
No	21	5	35	9	23	5	5	2	
Gender									
Female	239	52	214	52	209	47	133	42	0·02
Male	224	48	200	48	234	53	187	58	

** Missing data were noted for student-level characteristics regarding identifying as Aboriginal and/or Torres Strait Islander and primary language spoken at home.

* The ICSEA (Index of Community Socio-Educational Advantage) provides an indication of the socio-educational backgrounds of students.

† *P*-values calculated by Fisher’s exact test.

### School-level data

#### Teaching intervention

##### Feedback on training

Forty teachers completed the training. Seven teachers attended a face-to-face workshop with the remaining teachers completing the online training module (*n* 33). The online training received positive feedback, with each module being shown to increase knowledge. Teachers reported they were confident or very confident to deliver information to other year 7 and 8 teachers in their school (81·8 %), present the lesson content and activities to year 8 students (94·0 %), and support the implementation of the promotional aspects of the study within their school (75·7 %). On a scale of one to ten, teachers were asked how likely they were (with ten being highly likely) to recommend the online training, with 69·6 % ranking it as 7 or above. Suggestions for improvements included revising module length, ensuring content matched teachers experience and reviewing how materials within the online training platform downloaded as some content was too small.

##### Feedback on lesson component

**Fig. 2:**
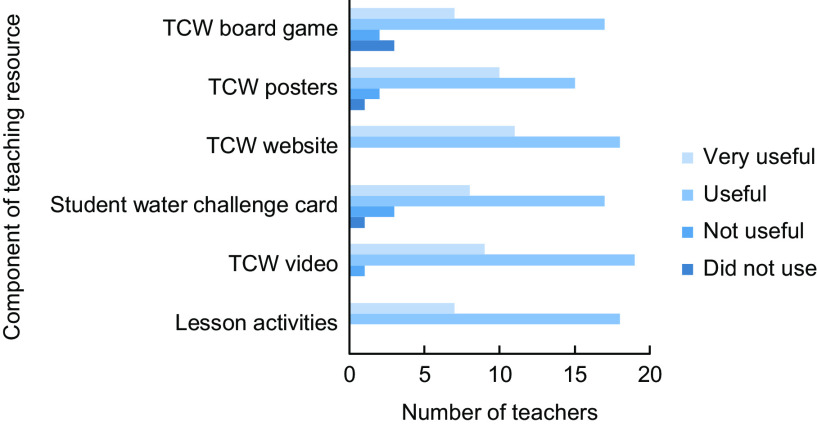
Usefulness of components of teaching resource. TCW, Thirsty Choose Water

Thirty-one PDHPE teachers completed the survey. The sample included eleven teachers from six schools across one LHD and twenty teachers from seven schools in another LHD. Of teachers who completed the survey, twenty-five completed the online training, four attended a face-to-face workshop, and two attended neither and were trained in the programme by a colleague who had attended the training. The time teachers spent on training ranged from 20 to 120 min. The average time spent on online training was 62 min. Most teachers (74 %; *n* 23) had only one year 8 PDHPE class to teach (range 1–4 classes). All teachers who delivered the lessons (*n* 29) delivered the programme to all their year 8 classes. Besides being trained in the programme, teachers spent an average of just under an hour (56 min) in lesson preparation (range 0–180 min). Compared with usual lesson time preparation, 55 % of teachers who delivered lessons reported it was ‘about the same’, whilst 28 % thought it was a ‘bit less’ and 21 % thought it was a ‘bit more’. Teachers either delivered the programme within the suggested number of lessons (two) or in three lessons (*n* 13 and *n* 14, respectively). Two teachers took a longer period to deliver the programme, doing this over four lessons. The average lesson time across these schools was 55 min, ranging from 40 to 60 min. Three teachers also used the lesson content with other year groups, delivering it to years 7, 9 and 10. Teachers who delivered the lessons (*n* 29) in general found most components of the teaching resources useful or very useful, as shown in Fig. 2. Data were missing for four teachers regarding the lesson activities.

Two-thirds of teachers reported they had taught similar content in the past, but it had been brief. All teachers intended to keep using the resources in the future.

While some teachers provided responses to open-ended questions, there was an insufficient quantity to enable a formal thematic analysis approach to these data. However, responses were scanned for positive or negative commentary and grouped into responses regarding lesson content and resources as well as the impacts of COVID-19. Regarding the programme resources and lesson content, all comments were positive, except for one which commented on the time it took to familiarise self with lesson content and resources, as shown below:

*‘All the resources provided assisted the delivery of the content and the students found it really beneficial’ (School 17, Group 3, LHD1).*

*‘The resources and teaching & learning activities provided, allowed for less time required to plan an engaging lesson for students’ (School 3, Group 1, LHD2).*

*‘The lesson plans and resources are excellent, greatly appreciated’ (School 5, Group 1, LHD1).*

*‘Familiarisation of each lesson and resources took time and to make adjustments for different learners’ (School 4, Group 1, LHD 1).*

The impacts of COVID-19 necessitated the shutdown of schools and a rapid pivot to online learning, with teaching materials swiftly amended to accommodate this. Teachers provided many positive comments about this, although this was challenging:

*‘Lessons were taught online using the support material provided by the HPO’ (School 13, Group 3, LHD2).*

*‘Parents loved the lessons on Zoom’ (School 15, Group 3, LHD2).*

*‘Lesson delivery was challenging due to students “off-site” completing online learning due to Covid-19’ (School 14, Group 3, LHD2).*

### Promotional intervention

**Fig. 3:**
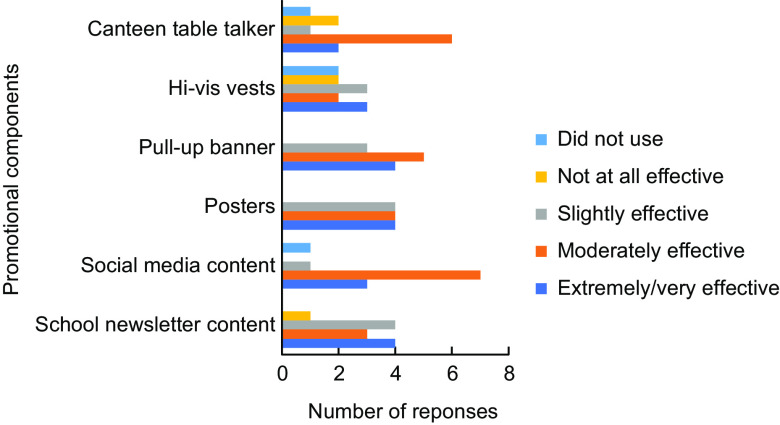
Effectiveness of promotional components

Schools in the BI reported on the effectiveness of the promotional resources as shown in Fig. 3. School newsletter content, posters and pull-up banners were seen to be extremely/very effective, whilst social media content and canteen table talkers were reported to be moderately effective. Schools also reported other ways they had promoted the programme, including promotion during monthly year meetings, the school principal raising the issue at assembly on numerous occasions and students developing promotional ideas.

### Environmental intervention

Schools in the environmental intervention had collected data on water usage through the CWS and changes in the promotion of water within the school from baseline to follow-up.

**Fig. 4:**
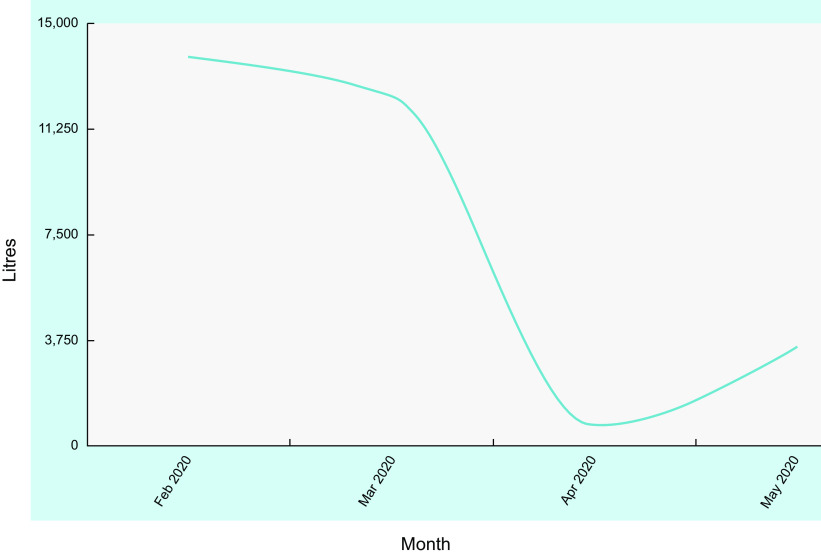
Water usage from chilled water stations

As previously described, data loggers were installed on CWS to monitor the flow of water through the station. Water flow data collected through the loggers in the initial study demonstrated an increase in CWS use over time and flow changes due to seasonality (greater flow in warmer months) and dependent on days of the week and weekends. For example, spikes in usage were noted on Tuesdays (consistent with sports days being commonly held on Tuesdays) and troughs on weekends when no students were on the school premises. However, within this current study, several issues with data collection through the loggers arose which impacted data quality. At several schools, the data loggers went offline at times for days (5–31 d). A combination of issues contributed to this – the distances required to travel to schools to address logger issues meant they were not resolved as quickly as they might have been and issues with internet reception meant data could not be collected. COVID-19 also affected CWS usage, as clearly shown in Fig. 4. There is a dramatic decline in usage at the end of March which remained low in April (school holidays) and May when most students were homeschooling.

Nevertheless, despite the issues with data loggers, water stations were still well received, with positive feedback from teachers and students alike. School staff commented that the CWS were well utilised, particularly given the warmer climates in some regions. Comments included:

*‘Something novel, it was extremely hot, so students were very appreciative of this´ (School 17, Group3, LHD1).*

*‘Excellent! The students and staff love it!’ (School 12, Group2, LHD1).*

*‘Students actively used it and were very pleased that it was installed’(School 7, Group2, LHD2).*

*‘Proved extremely popular with the students, especially during the hotter months. Great addition to the school.’ (School 11, Group2, LHD1).*

While there were some changes in the promotion of water in the schools, for example, promoting water on excursion notes and through sports, none were statistically significant within any of the schools (as shown in online Supplementary Table 1).

### Student-level data

Table 2 provides a within-group analysis across variables related to knowledge regarding the effects of SSB and water, water behaviours (including bottle and bubbler use) and consumption behaviours (SSB and water).

**Table 2:** Combined table of student-level data

		Group 1: Behavioural intervention					Group 2: Environmental intervention					Group 3: Combined intervention					Group 4: Control				
		Baseline *n* 462	%	Post *n* 292	%	*P*-value	Baseline *n* 414	%	Post *n* 268	%	*P*-value	Baseline *n* 463	%	Post *n* 318	%	*P*-value	Baseline *n* 320	*%*	Post *n* 290	%	*P*-value
Knowledge																					
Dehydration effects	I will get a headache	397	90	275	96	0·00	354	92	267	94	0·34	389	91	196	94	0·06	293	93	267	94	0·57
	I will feel thirsty	418	94	270	94	0·98	360	94	269	95	0·38	395	92	300	96	0·02	288	91	270	96	0·01
	I will feel energised	398	92	264	95	0·12	327	90	254	94	0·03	388	92	294	94	0·17	279	92	266	96	0·02
	I will have good concentration	397	92	258	94	0·22	335	92	256	96	0·10	372	89	282	92	0·20	281	93	270	97	0·02
	My skin will feel dry	281	65	244	85	0·00	260	70	209	75	0·11	299	71	261	84	< 0·00	209	68	215	77	0·10
	My wee will be dark	292	68	255	90	0·00	280	75	233	84	0·00	300	72	271	87	< 0·00	251	80	236	85	0·13
SSB effects	Weight gain	403	91	279	97	0·00	348	89	265	94	0·04	394	92	296	95	0·24	304	96	265	93	0·20
	Harm your teeth	426	96	285	99	0·04	368	95	280	99	0·01	408	96	310	98	0·04	310	98	275	99	0·42
	You must drink SSB after sport/exercise	395	88	268	93	0·03	352	91	272	95	0·02	389	91	295	94	0·12	295	93	270	95	0·37
	No nutritional value	261	59	181	64	0·22	219	57	187	66	0·01	253	59	180	57	0·59	212	67	165	59	0·02
Minutes walked to burn off can soft drink = 90 min		223	50	216	76	0·00	206	54	167	59	0·12	218	51	213	68	< 0·00	183	58	164	59	0·86
Which has more sugar	Soft drink, juice or the same	284	63	169	59	0·35	247	63	171	60	0·44	282	65	199	63	0·51	180	57	157	55	0·71
	Soft drink, sports drink or same	158	35	109	39	0·30	152	39	106	37	0·64	164	38	111	35	0·40	96	30	83	29	0·76
Water behaviours																					
Brings bottle	Never/not often	106	23	50	17	0·03	104	26	62	22	0·02	98	22	50	16	0·01	75	24	75	26	0·36
	Every day/mostly	350	77	239	83		297	74	223	78		340	78	266	84		244	76	212	74	
Fills bottle at school	Never	87	19	50	18	0·48	88	23	34	12	0·39	107	25	43	14	< 0·00	56	18	40	14	0·17
	Yes	362	81	235	82		303	77	242	88		325	75	269	86		253	82	239	86	
Drinks from bubbler	Never	89	19	69	24	0·09	57	14	52	18	0·06	96	22	101	32	0·00	24	8	37	13	0·01
	Yes	368	81	218	76		343	86	233	82		337	78	216	68		294	92	247	87	
Consumption behaviours																					
Sugar-sweetened beverages	Never/rarely	60	13	54	19	0·00	66	16	60	21	s 0·00	56	13	51	16	< 0·00	40	13	53	19	0·00
	1 cup or less/week	137	30	97	33		110	27	88	31		129	29	112	35		95	30	93	32	
	2–6 cups per week	170	37	98	34		136	33	96	34		165	37	122	38		118	37	90	31	
	1 cup or more daily	95	21	41	14		100	24	42	15		91	21	33	10		65	20	51	18	
Water	Never or rarely	9	2	3	1	0·18	4	1	4	1	0·02	5	1	5	2	0·06	1	0	4	1	0·03
	1 cup or less/week	11	2	2	1		12	3	3	1		11	2	3	1		6	2	4	1	
	2–6 cups/week	61	13	37	13		50	12	22	8		56	13	29	9		40	13	19	7	
	1 cup or more daily	378	82	247	85		342	84	255	90		367	84	280	88		268	85	262	91	

Students who received the BI demonstrated several positive changes from baseline to follow-up. There were several statistically significant changes in their knowledge regarding the effects of SSB, which appeared to translate to an increase in water bottle carrying behaviour, with 82 % reporting they bought a water bottle to school post-intervention compared with 76 % at baseline (*P* = 0·03). Pleasingly daily SSB consumption decreased from 20·56 % to 14·14 % (*P* < 0·01).

Students who received the environmental intervention only or the BI only showed a very similar pattern of results. They also had several statistically significant changes in their knowledge regarding the effects of SSB and showed changes in their bottle carrying behaviour (74·06 % pre; 78·25 % post; *P* = 0·02) and a decrease in daily SSB consumption (24·27 % pre; 14·69 % post: *P* < 0·01).

Students receiving both interventions showed statistically significant changes in their knowledge regarding the effects of SSB, and like the other groups, they increased their water bottler carrying behaviour. Students in this group, however, were also statistically more likely to fill up their water bottle at school (75·23 % pre; 86·22 % post; *P* < 0·01) (due to the CWS and new knowledge from the BI of the importance of water) and less likely to drink from the bubbler (77·83 % pre: 68·14 % post; *P* < 0·01).

Whilst group 4 results suggested some change (in a positive direction) in knowledge about the effects of dehydration, there was a statistically significant decrease in the proportion of students correctly identifying that SSB have no nutritional value, as well as a change in bubbler usage and decrease in daily SSB consumption.

There were decreases in the proportion of students reporting that they used the existing school bubblers (unchilled). However, this was only statistically significant for the schools receiving the combined intervention as well as control schools. There was an impact of COVID-19 as students were directed not to use the school bubblers and, in some schools, these were switched off, due to concerns about COVID-19 transmission.

From baseline to follow-up, the control group had less loss to follow-up for the student surveys than any of the intervention groups. This may be due to the control group having fewer year 7 students overall than any of the intervention groups, meaning that fewer year 7 PDHPE classes in these control schools, making the follow-up surveys easier to conduct. The control group also had a greater number of private schools and a higher Index of Community Socio-Educational Advantage (ISCEA) score for schools within the group compared with schools within the intervention groups. Group 1 had the greatest loss to follow-up, which could be related to the fact that this group had two schools that were either remote or outer regional, which may have affected connectivity to the internet. At one of the schools a teacher estimated that less than 50 % of students would have internet access, which would have impacted students’ ability to complete the survey if they had not done so before commencing home schooling due to COVID-19.

Whilst across all intervention groups, there was an increase in the proportion of students reporting drinking one cup or more of water daily at follow-up, this was only significant for the environmental intervention group and interestingly the control group. The proportion of students reporting SSB consumption of one cup or more daily decreased across all groups and was statistically significant.

To further examine changes and individual and joint effects of the interventions, multivariable ordinal logistic regression models were derived for each outcome (Table 3). Model adequacy tests showed that the model was appropriate, with the assumption of proportional hazards being satisfied and the model being specified correctly. As shown below, all interventions increased water consumption (though with no statistically significant effect). However, the odds of increased water consumption were higher for those students who received the environmental intervention only. Similarly, all interventions alone or combined decreased SSB consumption, but this decrease was only statistically significant for the environmental intervention (OR: 0·68; 95 % CI: 0·51, 0·90) and combined intervention (OR: 0·75; 95 % CI: 0·59, 0·97).

**Table 3:** Individual and joint intervention effects on the weekly consumption of water and SSB between baseline and follow-up

Intervention effect	Water consumption			SSB consumption		
	OR	95 % CI	*P*-value	OR	95 % CI	*P*-value
Behavioural intervention only	1·26	081, 1·96	0·31	0·79	0·61, 1·02	0·07
Environmental intervention only	1·32	0·79, 2·19	0·29	0·68	0·51, 0·90	< 0·01
Combined intervention	1·21	0·71, 2·03	0·49	0·75	0·59, 0·97	0·03

## Discussion

This study expanded the work of Thirsty? Choose Water! into regional and remote secondary schools within two NSW LHD. Where practicable, this study mirrored the parent study, but some changes to study implementation and evaluation were required, necessitated by funding timelines. Consequently, the vaccination intervention was not implemented, and student data were only collected at two time points, rather than three. The study also weathered challenges including the impacts of COVID-19 and issues that arose due to school locality. Nevertheless, these regional and remote schools achieved similar levels of acceptability and outcomes as the parent study.

Within schools that received the BI, there was positive feedback from teachers on the training to deliver the intervention and the intervention components themselves. Eighty-six per cent of teachers reported the lesson content to be useful to very useful, with all teachers reporting the Thirsty? Choose Water! website to be ‘useful’ to ‘very useful.’ The environmental intervention also had a high level of acceptability. Within these schools, staff reported that the CWS were well utilised and appreciated by students, particularly in hot weather. Schools within the study also made some changes to their policies regarding water, such as including information about bringing water on excursion notes and promoting water within sports and school newsletters.

Regarding student outcomes, the within-group analysis demonstrated several statistically significant changes in knowledge, water behaviours and consumption. Students receiving the combined intervention had a statistically significant change from baseline to follow-up on water behaviours, such as bringing a bottle to school, filling it up at school and drinking from the bubbler. Students in both the environmental intervention and control reported a statistically significant increase in water consumption. Students across all groups showed a significant decrease in SSB consumption. Statistically significant changes were also noted in several variables for the control group, which could be explained by the hawthorn effect, whereby the very nature of being involved in a study may influence behaviour change. The smaller sample size in this study may also not have had the power to withstand these sorts of effects.

Like the parent study, this study used logistic regression to examine the impacts of the individual and combined effect of the interventions on both water and SSB consumption. Like the parent study, these outcomes showed that odds for increasing water consumption or reducing SSB consumption all moved in a positive direction, although only some were statistically significant. In terms of decreasing SSB consumption, both the environmental intervention alone and the combined interventions had a statistically significant effect. However, none of the interventions (either alone or combined) produced a statistically significant effect on increasing water consumption.

This study adds to a growing literature regarding interventions in school settings to increase water consumption and decrease SSB consumption among students. To effectively address this issue, there are calls for multicomponent interventions in schools that address the environment, the individual or both^(18–21)^. Moreover, more rigorous study designs such as randomised controlled trials which report more specifically on intervention effects are required^(25)^. This current study meets both of these with its randomised design, the implementation of a multicomponent intervention and the evaluation of specific intervention, including changes in consumption of SSB and water. Our findings add weight to earlier evidence that school-based water consumption interventions can displace SSB^(38)^ as evidenced by our finding that the environmental intervention alone decreased SSB consumption. Nevertheless, interventions focusing on both the environment and the curriculum are likely warranted, given in our study the greater effect on decreasing SSB consumption for the combined group.

There were several challenges for this study, with the impacts of distance, natural disasters and COVID-19 combined impacting study implementation and evaluation. While the regionality and remoteness of schools did not impact the acceptability of the interventions, it did present some issues for the rigour of the evaluation of the research and its outcomes. For example, the utility of the data loggers as an objective measure of water consumption within this study is questionable and cannot be used to its full potential. This was because in some regional/remote locations, schools did not have good internet connectivity. This meant that data loggers were often ‘offline’, being unable to connect to the internet and log data. Therefore, the dataset is incomplete. Similarly, internet connectivity impacted other aspects of the study evaluation to some degree.

At the commencement of the study (Spring 2019; September–November), bushfire emergencies arose in both study regions. This impacted the usual running of some schools and had a knock-on effect on study implementation, delaying baseline data collection for some schools and therefore CWS installation. The emergence of COVID-19 in early 2020 also presented further challenges to study. Although most schools in the BI groups had delivered the programme before the first COVID-19 lockdown in March 2020, some schools had not. Subsequently, the project team acted swiftly to ensure that the programme could be delivered online whilst students were homeschooling. Although teachers provided positive feedback about this, we are aware that some students had issues with accessing online platforms and internet connectivity. This is consistent with other research that examine the impact of COVID-19 on the delivery of health promotion interventions in schools and some students’ inability to connect with the remote learning aspects^(39,40)^. This issue also impacted the collection of student data follow-up, and this may have impacted evaluations outcomes. While the baseline data collection was predominantly completed in a classroom with oversight from the teacher, this was not necessarily the case for the follow-up data. Students were provided with the link to the survey to complete it, but the oversight of this happening was limited as students were not physically in the classroom. This could explain why the loss to follow-up was substantial for some schools. While our analysis used all available data to inform parameter estimates by the avoidance of a complete-case analysis, further research concerning the missing mechanism is recommended to determine if our estimates have been unduly impacted by missing data.

The study’s strengths are that it extended both an intervention and study design that had been implemented effectively previously. One limitation of the parent study was the inability to detect changes in water consumption due to the blunt nature of the Likert scale that gathered that data. In this study, we attempted to overcome that, even with limited success. While we asked about water consumption as a continuous variable, we could not incorporate that data field into the logistic regression, as the SSB consumption had been measured as a categorical variable. Therefore, despite attempts to address this limitation, we had to revert to the categorical data for water consumption when it came to include variables into the logistical regression.

Overall, the results of this study continue to mirror the findings of the parent and pilot studies. That being that schools are willing and ready to address this issue, that it aligns well within their PDHPE curriculum and that they appreciate access to chilled water. Regarding water and SSB consumption outcomes, the interventions appear to have had a positive effect in the direction of the desired change. This would warrant further investigation into the translation of intervention components more broadly into school settings. To support this in secondary school settings in NSW, a range of Thirsty? Choose Water! resources were developed and can be accessed at https://www.choosewater.com.au/. Gamification has also been used to draft the messages into an online game Aqua Sprint. Other research has shown the utility of gamification for sharing health messages about this issue^(41)^. The study also has policy implications. Mapping the intervention components into the PDHPE curriculum encourages implementation and through the provision of lesson content allows for greater intervention fidelity – this would suggest that the intervention is scalable, as it links to state-wide. Moreover, the intervention can impact school-level policies to promote water, with schools in both this and the parent study readily able to make changes to promote water on excursion notes during sports and to allow students to have water bottles on their desks during class. Secondly, both this and the parent study highlight the benefit and desirability of CWS in schools. While the study utilised the ratio of one CWS per 300 students, there was often feedback that the stations were so popular that students were lining up for water. Further work is required to inform policy makers at both state and national levels of this need, which can then be used to inform installation of water stations at scale within secondary schools. Economic evaluations of the study interventions are pending, with the intention that these will further inform policy decisions and facilitate action on the need to provide chilled water in secondary schools and education about the detrimental effects of SSB.

## Supplementary material

For supplementary material accompanying this paper visit https://doi.org/10.1017/S1368980023001313. click here to view supplementary material
