# Supplementary material for: Thirsty? Choose Water! A regional perspective to promoting water consumption in secondary school students
Source: Public Health Nutr. 2023 Jul 10;26(11):2526–38. doi: 10.1017/S1368980023001313 (PMC10641618; doi:10.1017/S1368980023001313)
Supplement: Supplementary file 1 [file S1368980023001313sup001.docx]

**Supplementary tables**

*Table 1:* ***School-level data*** *Promotion of water within schools*

|  | **Group 1**  **Behavioural intervention** | | | **Group 2**  **Environmental intervention** | | | **Group 3**  **Combined intervention** | | | **Group 4**  **Control** | | |
| --- | --- | --- | --- | --- | --- | --- | --- | --- | --- | --- | --- | --- |
| **Water promotion activity** | Baseline  (T1)  n=6 | Post (T2)  n=6 | P-value | Baseline  (T1)  n=6 | Post (T2)  n=6 | P-value | Baseline  (T1)  n=6 | Post (T2)  n=6 | P-value | Baseline  (T1)  n=6 | Post (T2)  n= 6 | P-value |
| Water on desks | 6 | 6 | ns | 5 | 6 | ns | 5 | 5 | ns | 6 | 5 | ns |
| Excursion notes | 6 | 6 | ns | 3 | 5 | ns | 2 | 6 | ns | 3 | 4 | ns |
| Promoted in sport | 6 | 6 | ns | 3 | 6 | ns | 3 | 5 | ns | 5 | 4 | ns |
| Newsletter | 2 | 4 | ns | 0 | 4 | ns | 2 | 6 | ns | 1 | 3 | ns |

ns – not significant
